# Diffusion MRI-based analysis of functional alterations of the glymphatic system in children with non-lesional epilepsy

**DOI:** 10.1016/j.ynirp.2025.100237

**Published:** 2025-01-30

**Authors:** Bixia Wu, Gengbiao Zhang, Yanting Wang, Hongyi Zheng, Hui Tan, Wenbin Zheng

**Affiliations:** a Department of Radiology, Meizhou People's Hospital, Meizhou, China; b Department of Radiology, The Second Affiliated Hospital, Shantou University Medical College, Shantou, China

**Keywords:** Children, Non-lesional pediatric epilepsy, Glymphatic system, DTI-ALPS, Free-water mapping, MRI

## Abstract

**Objective:**

Based on diffusion tensor imaging (DTI) data, we used DTI-analysis along the perivascular space (DTI-ALPS) and free-water mapping to investigate the function of the glymphatic system and its relationship with clinical features among pediatric patients with magnetic resonance imaging (MRI)-negative non-lesional epilepsy (NLE).

**Methods:**

A total of 83 NLE children (mean age 9.25 ± 4.07 years) and 45 matched healthy controls (mean age 9.36 ± 3.89 years) were recruited. All eligible patients were routinely scanned by 3.0T MRI to rule out organic lesions, and DTI data were collected at the same time. The ALPS index and fractional volume of free water in white matter (FW-WM) in the brain were calculated to analyze the differences between groups and the correlation between the corresponding parameters and clinical indicators such as age of onset, duration of the disease, seizure frequency, and seizure duration.

**Results:**

The NLE group had significantly lower ALPS indexes in the left (ALPS-L, F = 4.415, p = 0.038) and right (ALPS-R, F = 12.673, p = 0.001) cerebral hemispheres compared to the HC group. ALPS-L was positively correlated with age of onset (r = 0.322, p = 0.008) and negatively correlated with FW-WM (r = −0.337, p < 0.001). Free-water mapping revealed substantially higher FW-WM in the NLE group compared to the HC group (F = 4.666, p = 0.033). Additionally, FW-WM was negatively correlated with age of onset (r = −0.463, p < 0.001) and positively associated with seizure control in children with NLE (r = 0.306, p = 0.012).

**Conclusion:**

Children with NLE have reduced glymphatic system function, and the underlying pathophysiologic mechanisms may be due to impaired interstitial fluid clearance and retention in the brain. DTI-ALPS and free-water mapping are useful noninvasive approaches for examining glymphatic function in children with NLE, with the FW-WM potentially serving as an imaging marker for disease progression and predicting clinical prognosis in children with NLE.

## 1 Introduction

As a neurodegenerative disorder, epilepsy is characterized by the presence of unprovoked and recurrent seizures. Its conclusive diagnosis is based on a variety of procedures, including clinical assessment, electrophysiological testing, and imaging examinations. Although detection of epileptic foci has been significantly improved with the development and popularization of magnetic resonance imaging (MRI) technology, the abnormal brain structures of some patients still cannot be accurately identified, which is then considered to be non-lesional epilepsy (NLE). According to statistics, incidence of NLE is increasing, accounting for nearly two-thirds of all cases of epilepsy (ZHANG et al., 2011). However, it is worth noting the abnormal brain structures and the source of epileptiform discharges among NLE patients are not easily or accurately to be identified in traditional imaging examinations such as computed tomography (CT) and MRI, which brings difficulties in clinical diagnosis and treatment.

Epilepsy may be genetically determined or acquired, which occurs following epileptogenic events such as traumatic brain injury, status epilepticus, infection, or stroke (PITKäNEN and LUKASIUK, 2011). The development of epilepsy is likely multifactorial and lymphatic system damage may be one of them. The excessive glutamate release and increases in oxidative stress and inflammatory mediators during seizures can trigger damage to the blood-brain barrier and interfere with the normal flow of cerebrospinal fluid to interstitial fluid (CSF-ISF) as well, thereby affecting glymphatic system function (LIU et al., 2020; GORTER et al., 2015). In 2012, Illiff and Nedergaard et al. first observed the exchange of CSF with ISF in the mouse brain using two-photon imaging and named this process the glial-lymphatic system (ILIFF et al., 2012), which was also called glymphatic system. Its mechanism of action involves penetration of CSF into brain tissue along the periarterial space and exchange with ISF via aquaporin 4 (AQP4), which drives metabolic waste products and ISF into the perivenous space and eventually returns to CSF circulation, or is excreted through cervical lymphatic capillaries. This system plays a key role in transporting nutrients and immune cells, as well as removing waste products (RASMUSSEN et al., 2018).

Further study has indicated that epilepsy may lead to blood-brain barrier dysfunction in pediatric patients, which affects their normal operation of glymphatic system (DADAS et al., 2016). Seizures may lead to abnormal accumulation of tau protein, thus destroying the circulation of glymphatic system and resulting in further seizures or persistent epilepsy (GORTER et al., 2015). In addition, vascular responses following seizures can interfere with glymphatic system function (GIROUARD and IADECOLA, 2006). Diffusion tensor imaging analysis along the perivascular space (DTI-ALPS) is a technology proposed by Japanese scientist Taoka et al. (TAOKA et al., 2017) in 2017 that assesses the activity of the glymphatic system by measuring the diffusion rate of water molecules in the perivascular space. It has the advantage of reflecting the glymphatic system's clearance function(LIU et al., 2024). Free-water mapping can precisely separate and correct free-water components of the brain, indirectly reflecting the "water storage status" of the glymphatic system(HOY et al., 2014). The combination of ALPS and free-water mapping can provide a more comprehensive evaluation of the glymphatic system's operational state in NLE patients(LIN et al., 2024). Previous studies have confirmed glymphatic system dysfunction in epileptic children (LEE et al., 2022a; LEE et al., 2022b; LEE et al., 2022c; PU et al., 2023), but very few studies have focused on children with NLE (LEE et al., 2022a). Therefore, quantitative analysis of glymphatic system function among NLE children will shed light on the cause and treatment selection of epilepsy.

By conducting comparison of ALPS index and fractional volume of free water in white matter (FW-WM) between children with NLE and healthy controls, we aimed to investigate the glymphatic system function of NLE children and to figure out the potential association between glymphatic system dysfunction and clinical characteristics.

## 2 Materials and methods

### 2.1 General information

#### 2.1.1 Study population

The institutional review board of the Second Affiliated Hospital of Shantou University Medical College approved this retrospective study and all patients' family members signed the informed consent. A total of 83 NLE children (mean age 9.25 ± 4.07 years) and 45 gender- and age-matched healthy controls (mean age 9.36 ± 3.89 years) from August 2021 to September 2023 in our hospital were recruited. All eligible patients were routinely scanned by 3.0T MRI to rule out organic lesions, and DTI data were collected at the same time. Clinical characteristics included education, age of onset, age at examination, duration of disease, seizure frequency and seizure duration. Age of onset was defined as the age of the first seizure, age at examination was defined as the actual age of hospital visit this time, and duration of disease was defined as the length from onset age to this examination. The number, frequency, duration, and other clinical manifestations of seizures were dictated by patient's family, telephone interview, or inpatient medical records. Three subgroups were identified (KANEMURA et al., 2018): well-controlled epilepsy (WCE) (no seizures in the past year), uncontrolled epilepsy (UCE) (average of more than 1 seizure in the past year) and intermediate-controlled epilepsy (ICE) (average of 1 and less seizure in the past year).

#### 2.1.2 Inclusion and exclusion criteria

The inclusion criteria were as follows: (1) confirmed epilepsy according to ILAE 2017; (2) no intracranial lesions from routine MRI; (3) younger than 17 years of age; (4) right-handed. Patients were excluded if (1) they had contraindications of MRI, such as inability to cooperate and metal implantation; (2) they had history of intracranial surgery or were combined with other central nervous system diseases; (3) they had organic lesions according to the routine MRI, such as intracerebral tumors, hippocampal sclerosis, or encephalitis; (4) there had artifacts or excessive head movement in MRI results; (5) they were left-handed; (6) they were treated with chloral hydrate drugs for sedation.

### 2.2 Imaging acquisition

A GE 3.0-T MRI system (Signa; General Electric Medical System, USA) with a 8-channel head coil was used for regular and special sequence imaging scans. During the scanning process, participants were guided to lie supine with their heads immobilized so as to limit head motions. Also, they were accompanied by a guardian throughout so that the scan could be discontinued quickly in the event of an emergency. The diffusion imaging data acquisition parameters are as follows.

DTI-ALPS data: Conducted using spin-echo single-shot echo-planar pulse sequence in 30 diffusion directions, TR/TE = 8000 ms/90.2 ms, slice thickness = 2.5 mm, no gap between slices, FOV = 24 cm∗24 cm, acquisition matrix = 128∗128, b = 0 and 1000 s/mm^2^. A total of 899 images were obtained.

Free-water mapping data: Conducted using spin-echo single-shot echo-planar pulse sequence in 15 diffusion directions, TR/TE = 8000 ms/99.3 ms, slice thickness = 4 mm, no gap between slices, FOV = 24 cm∗24 cm, acquisition matrix = 128∗128, b = 0 and 1000 s/mm^2^. A total of 480 images were obtained.

### 2.3 DTI-ALPS processing

**Fig. 1:**
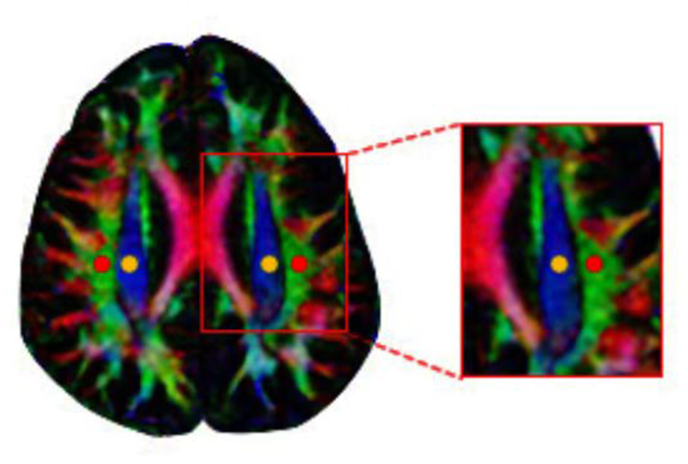
ROIs with a diameter of 5 mm in the projection fibers (yellow) and association fiber (orange) at the level of the lateral ventricle body.

DTI data were converted into NIFTI format using the dcm2niigui.exe program. DTI Studio software was used to construct diffusion tensor maps from NIFTI files, in order to obtain color-coded fractional anisotropy (FA) map and the tensor map. From each tensor image, diffusivity was automatically calculated in x-, y- and z-axis. In the lateral ventricle body, perivascular space is usually perpendicular to the projection and association fibers of x-axis, association fibers of z-axis and projection fibers of y-axis, so calculating the corresponding diffusion rate can reflect the diffusion of perivascular space. On the FA map at the level of the lateral ventricle body (Fig. 1), region of interest (ROI) (diameter = 5 mm) were plotted in the projection fibers and association fibers of the left and right hemisphere, from which diffusivities in the x-, y- and z-axis for these fibers were obtained. ROIs were determined independently by two neuroimaging physicians with extensive data processing experience, who were asked to keep the ROIs of diverse data in the same location. To eliminate the effect of subjective awareness on the measures, we did not inform the physicians in the group to which the measurements were related. Both were asked to measure the ALPS index three times, and the average of their results was taken as the final output.

ALPS-index was calculated to assess brain clearance pathways and the formula with diffusivity ratio in 4 different directions was as follows according to Taoka et al. (TAOKA et al., 2017):

$$ALPS\;\text{index}=\frac{Mean\left(D_{xx,proj},D_{xx,assoc}\right)}{Mean\left(D_{yy,proj},D_{zz,assoc}\right)}$$

Dxxproj and Dxxassoc refers to the diffusivity along the x-axis in the projection and association fiber, respectively. Dyyproj and Dzzassoc refers to the diffusivity along the y-axis in the projection fiber and along the z-axis in the association fiber, respectively.

### 2.4 Free-water mapping processing

Panda software (https://www.nitrc.org/projects/panda/) was used to preprocess DTI data, with procedures such as brain extraction, eddy current correction, and head motion correction. The FW-WM, as well as the free water-corrected mean diffusivity (MDt), fractional anisotropy (FAt), radial diffusivity (RDt), and axial diffusivity (ADt), were retrieved using Hoy et al.'s two-compartment model(HOY et al., 2014). The extraction was carried out using the open-source software program Diffusion Imaging in Python (Dipy) technique (https://dipy.org/). The extracted images were then analyzed using TBSS, which included preprocessing each subject's FA image to exclude potential outliers from the diffusion tensor fit. All subjects' FA images were aligned to standard space (FMRIB58_FA) using the FMRIB nonlinear alignment program. The aligned FA images were averaged to produce 4D FA images. The FA data from every single subject were projected onto the mean FA skeleton. Based on the statistical preparation of voxels, the mean FA skeleton imagine was thresholded with a value of 0.2, and the resulting binary skeleton mask specified the set of voxels used in subsequent processing. The mean values of whole-brain white matter FW-WM, FAt, ADt, RDt, and MDt were derived for each subject based on the mean fiber skeleton mask.

### 2.5 Statistical analysis

Shapiro-Wilk (S-W) test was first performed for normal distribution and homogeneity of variance test. Continuous variables were calculated as mean ± standard deviation (M±SD) or median and interquartile range (p25, p75) for normally distributed and non-normally distributed ones, separately. Numerical differences between groups were assessed by *t*-test or Mann-Whitney *U* test and correlation analysis was conducted by Pearson correlation test or Spearman rank correlation test. Gender difference was identified by chi-square test. The threshold for significance was set at *P* < 0.05. All statistical analyses were conducted using SPSS, Version 24.0.

The values of each imaging parameter (ALPS, FW-WM, FAt, ADt, RDt, MDt) were analyzed using One-Way ANCOVA, with the age as a covariate.The values of FW-WM, FAt, ADt, RDt, and MDt at the voxel level were compared using the FSL software's nonparametric randomization tool. The randomized permutation test was used to compare individual parameters in the standardized space of the two groups of data (5000 permutations), with age as a covariate. A difference was considered statistically significant at *P* < 0.05, and a correction for multiple comparisons was made using the Threshold Free Cluster Enhancement (TFCE). The anatomical locations of significant brain regions were determined using the XTRACT atlas(WARRINGTON et al., 2020).

## 3 Results

### 3.1 General information

Six controls and sixteen NLE cases were eliminated because of excessive head movements, poor image quality, or the use of sedatives including chloral hydrate. As shown in Table 1, a total of 67 NLE and 39 matched healthy controls were finally included, without significant inter-group differences in age, gender and education level (*P* > 0.05).

**Table 1:** Comparison of demographics between NLE group and healthy control group.

	NLE group	HC group	*t/χ*^2^ value	*P* value
Number of included cases	67	39	–	–
Sex (man/woman)	35/32	18/21	0.56	0.46^a^
Age at examination (year)	9.55 ± 4.08	9.92 ± 3.82	−0.47	0.64^b^
Age of onset (year)	7.52 ± 4.30	–	–	–
Duration of disease (month)	23.82 ± 39.39	–	–	–
Seizure frequency (number)				
WCE/ICE/UCE	4/41/22	–	–	–
Seizure duration (minute)	14.80 ± 40.75	–	–	–
Education (year)	3.29 ± 2.34	4.13 ± 3.53	−0.27	0.79^b^

*Notes:* WCE = well-controlled epilepsy (no seizures in the past year); UCE = uncontrolled epilepsy (average of more than 1 seizure in the past year); ICE = intermediate-controlled epilepsy (average of 1 and less seizure in the past year). ^a^ and ^b^ represents the use of chi-square test and two independent sample *t*-test, separately. The threshold for significance is *P* < 0.05.

### 3.2 Analysis of left and right ALPS-index

**Fig. 2:**
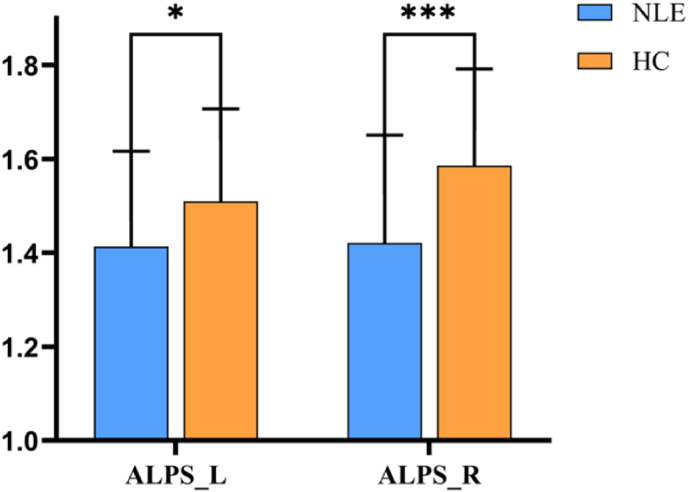
Comparison of ALPS index in the bilateral cerebral hemispheres of the NLE and HC groups. ∗ indicates *P* < 0.05, ∗∗∗ indicates *P* < 0.001. Abbreviation: ALPS: Analysis along the perivascular space; NLE: Non-lesional epilepsy; HC: Health control.

The main effect showed that the presence of epilepsy had a significant effect on the ALPS index of the subjects after taking into account the effect of the covariate of the age of the subjects, which was significantly lower in both the left and right brain in the NLE group compared to the HC group (ALPS-L, F = 4.415, p = 0.038; ALPS-R, F = 12.673, p = 0.001). The covariate effect showed that age was a significant covariate for the ALPS-L index (F = 13.517, p < 0.001), suggesting that the age factor had a significant effect. There was no statistically significant difference between ALPS-L and ALPS-R index (p = 0.870). (Fig. 2).

### 3.3 Free-water mapping analysis

Under the comparison of whole brain white matter levels, the main effects showed that the presence of epilepsy had a significant effect on subjects' FW-WM after accounting for the effect of age as a covariate, which was significantly higher in the NLE group compared to the HC group (F = 4.666, p = 0.033), but there was no significant effect on FAt (F = 1.089, p = 0.299), MDt (F = 0.801, p = 0.373), ADt (F = 0.420, p = 0.519), and RDt (F = 0.626, p = 0.431). The covariate effect showed that age was a significant covariate for FW-WM (F = 36.762, p < 0.001), FAt (F = 52.668, p < 0.001), MDt (F = 65.762, p < 0.001), ADt (F = 64.647, p < 0.001), and RDt (F = 63.573, p < 0.001).

**Fig. 3:**
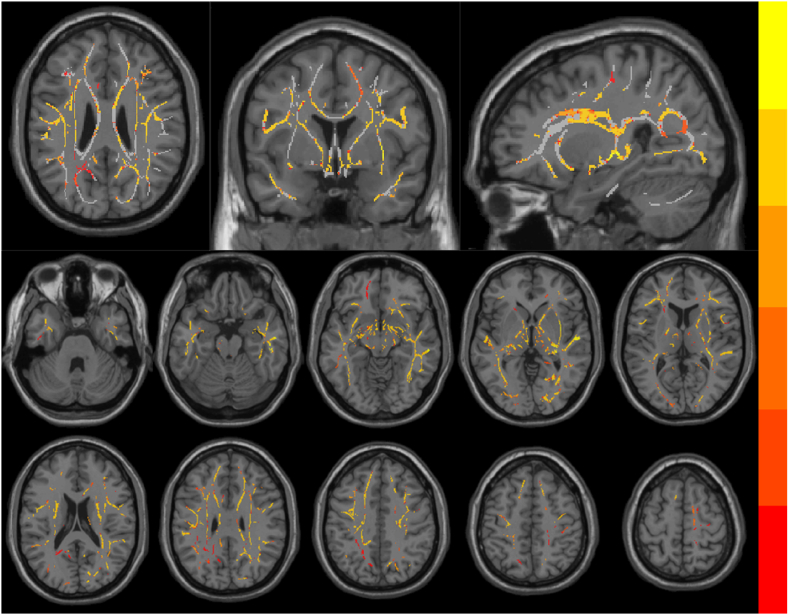
Brain region distribution of FW-WM difference; grey areas represent the mean FA fiber skeleton, and color bars show the significance of FW-WM difference in the respective brain regions.

Under voxel level comparison, the NLE group had significantly higher FW-WM in 15 clusters (Fig. 3); for further information on the clusters, please refer to Supplementary Table S1, and there were no significant differences in the other parameters.

### 3.4 Correlation analysis

**Fig. 4:**
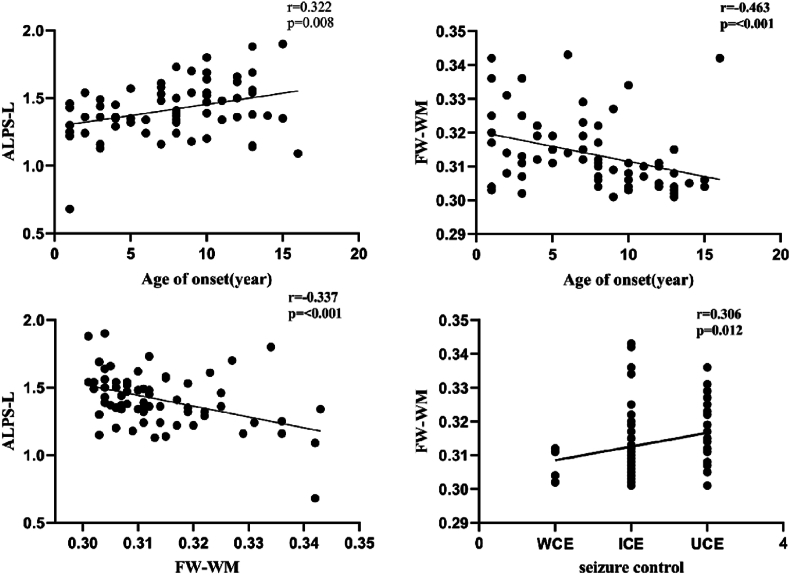
Correlation of ALPS index and FW-WM with clinical features in NLE group. Abbreviation: WCE, well-controlled epilepsy (no seizures in the past year); ICE, intermediate-controlled epilepsy (average of 1 and less seizure in the past year); UCE, uncontrolled epilepsy (average of more than 1 seizure in the past year). r represents correlation coefficient.

The ALPS-L index of the NLE group showed a positive correlation with age of onset (r = 0.322, p = 0.008) and a negative correlation with FW-WM (r = −0.337, p < 0.001). FW-WM showed a negative correlation with age of onset (r = −0.463, p < 0.001) and a positive correlation with seizure control in children with NLE (r = 0.306, p = 0.012) (Fig. 4). There was no statistically significant association between the ALPS-R index and any clinical features (p > 0.05) (Table 2.).

**Table 2:** Correlation of ALPS index and FW-WM with clinical features in NLE group.

	NLE Group					
	ALPS-L index		ALPS-R index		FW-WM	
	*r value*	*P value*	*r value*	*P value*	*r value*	*P value*
Age of onset (year)	**0.322**	**0.008∗∗**	0.199	0.106	**−0.463**	**<0.001∗∗∗**
Duration of disease (month)	−0.030	0.807	−0.194	0.117	−0.162	0.191
Seizure duration (minute)	0.029	0.834	0.173	0.208	−0.056	0.687
Seizure frequency (WCE/ICE/UCE)	−0.068	0.586	0.006	0.963	**0.306**	**0.012∗**
FW-WM	**−0.337**	**<0.001∗∗∗**	−0.094	0.339	–	–

*Notes:* NLE = non-lesional epilepsy. WCE = well-controlled epilepsy (no seizures in the past year); UCE = uncontrolled epilepsy (average of more than 1 seizure in the past year); ICE = intermediate-controlled epilepsy (average of 1 and less seizure in the past year). *r* represents correlation coefficient; ∗, ∗∗ and ∗∗∗ represents *P* < 0.05, *P* < 0.01 and *P* < 0.001, separately.

## 4 Discussion

As epilepsy and glymphatic system becoming the research focus, a growing body of evidence has revealed their association. Research has shown that patients with epilepsy have impaired metabolic waste elimination in their ISF and disturbed CSF flow. (MARCHI et al., 2016). It has been pointed out that seizures may damage the blood-brain barrier, divert and accumulate solutes in serum into the brain, and alter the ionic balance of ISF, thereby causing cerebral edema(OBY and JANIGRO, 2006). These long-term pathological changes possibly lead to dynamic changes in fluid in the brain, reduced efficiency of clearance mechanisms, and accumulation of harmful substances as well. In this study, we evaluated the glymphatic system function in children with NLE using the brain's ALPS index and FW-WM. We discovered that the children's ALPS index decreased while their FW-WM increased, and that the corresponding parameters were associated with seizure control and the age at which the disease began. These findings help us better understand the pathophysiological mechanisms of children with NLE.

According to DTI-ALPS analysis, children with NLE had significantly lower ALPS indices in both the left and right brains than the HC group. This suggests that children with epilepsy who did not exhibit any abnormal changes on MRI may have a widely impaired glymphatic system clearing function. On the other hand, the current study also discovered that FW-WM was elevated in both cerebral hemispheres. FW-WM is known to assess the content of ISF in the brain, and correlation analysis revealed that the ALPS-L index was negatively correlated with FW-WM. This suggests that a greater accumulation of ISF would result from a more impaired glymphatic system clearance, so we hypothesized that impaired glymphatic system clearance, which leads to impaired ISF drainage, could be one of the causes of the increase in FW-WM. In an MRI study of glymphatic function in children with Rolandic epilepsy, Yin et al. came to the same conclusion. Previous studies on different subtypes of epilepsy indicated that juvenile myoclonic epilepsy (JME) (LEE et al., 2022b), status epilepticus (LEE et al., 2022c) and initially diagnosed focal epilepsy (LEE et al., 2022a) were associated with glymphatic system dysfunction, but most studies ignored the effect of the lesions. Consistent with prior studies, our study found glymphatic system dysfunction in NLE children, thus we hypothesized that it might be a pathophysiological feature prevalent in patients with epilepsy and not limited to any specific type.

Furthermore, in right-handed NLE patients, glymphatic system dysfunction was not solely in the left hemisphere, but the right hemisphere showed a greater decreasing trend compared with the healthy controls. The correlation analysis found a positive link between ALPS-L index and age of onset in children with NLE, which could mean that the later the disease develops, the less impairment of the left cerebral glymphatic system function they already have. The above result was similar with Zhao et al. (ZHAO et al., 2023) who noted that adult patients with temporal lobe epilepsy presented asymmetries in glymphatic system function, with more severe impairment in the diseased side than in the healthy contralateral side. These findings all highlighted the potential functional asymmetry in brain regions among NLE children. In addition, the correlation analysis between ALPS-R index and each clinical characteristic was not statistically significant, implying that for right-handed children with NLE, the left brain may play a more critical role in the post-compensation of the functional impairment of the glymphatic system associated with their epilepsy, whereas the right brain may have a lesser influence or a different mechanism. This difference could be related to functional lateralization, in other words, differences between the left and right brain in processing information and maintaining network connectivity.

Notably, correlation analysis revealed that FW-WM was positively associated with seizure control in children with NLE, implying that greater ISF may indicate poorer seizure control. The current study suggests that seizures may be linked to impaired water clearance caused by AQP dysfunction, specifically AQP4(BONOSI et al., 2023), and Ayushe et al. (SHARMA et al., 2023) and Isabella et al. (GIACHETTI et al., 2022) have also found evidence of increased free water in the onset brain region in studies of patients with temporal lobe epilepsy. Although the particular mechanisms by which increased free water induces seizures and influences the efficacy of epileptic medication action are not entirely understood, its relationship with seizure control is still worth noting. From this perspective, FW-WM outperforms the ALPS index as a prospective imaging marker for monitoring disease progression and predicting clinical outcome in children with NLE.

All above results may be related to the pathophysiological mechanism of epilepsy, but the exact cause of glymphatic system dysfunction in patients with epilepsy remains inconclusive. Previous studies have mainly investigated the effects of factors involving inflammation (GORTER et al., 2015), sleep issues (Reddy and VAN DER Werf, 2020), AQP4 abnormalities (LOHELA et al., 2022), glutamate excess, and glutamate receptor hyperactivation (GORTER et al., 2015; DADAS et al., 2016) after epilepsy. In addition, given that glymphatic system includes glial cells, the phenomenon of increased activated microglia in epilepsy (ALTMANN et al., 2022) may support the dysfunction. Research of Zhang et al. (ZHANG et al., 2021) and Liu et al.(LIU et al., 2024) also confirmed the reliable consistency and robustness of ALPS across repeated measures. Although the ALPS index still has problems such as low interpretability, small measurement range, and subjective ROI outlining (TAOKA et al., 2024; HALLER et al., 2024; RINGSTAD, 2024), combining it with free-water mapping or perivascular space volume measurement can weaken its shortcomings to some extent and provide more comprehensive biological information for the study of epilepsy patients' glymphatic function, and combining it with different types of MRI techniques is highly valuable as a potential research direction (LIN et al., 2024; YIN et al., 2024).

## 5 Conclusion

Children with NLE have reduced glymphatic system function, and the underlying pathophysiologic mechanisms may be due to impaired ISF clearance and retention in the brain. DTI-ALPS and free-water mapping are useful noninvasive approaches for examining glymphatic function in children with NLE, with the FW-WM potentially serving as an imaging marker for disease progression and predicting clinical prognosis in children with NLE.

## 6 Innovations

Current studies have seldom focused on glymphatic system among pediatric patients with MRI-negative NLE. Based on ALPS index and FW-WM, this study innovatively investigated the relationship between glymphatic system function and clinical characteristics, providing imaging evidence for the pathophysiological process of brain in children with epilepsy.

## 7 Limitations and prospects

Given that the several types of epilepsy, this study did not make a further distinction between its different subtypes and age, but mainly concentrated on group research of NLE and normal controls. Studies in the future should conduct personalized analysis and judgment, and verify our results among different types and patients. In addition, patients might have history of antiepileptic drugs at the time of the MRI examination, and their potential impact on glymphatic system function were not ruled out in this study.

## CRediT authorship contribution statement

**Bixia Wu:** Writing – review & editing, Writing – original draft, Visualization, Validation, Investigation, Data curation, Conceptualization. **Gengbiao Zhang:** Writing – review & editing, Software, Methodology, Formal analysis, Conceptualization. **Yanting Wang:** Visualization, Validation, Investigation, Formal analysis. **Hongyi Zheng:** Supervision, Methodology, Investigation, Conceptualization. **Hui Tan:** Visualization, Methodology, Data curation. **Wenbin Zheng:** Supervision, Resources, Project administration, Funding acquisition, Conceptualization.

## Declaration of competing interest

The other authors have no conflict of interest to disclose.

## Data Availability

Data will be made available on request.
