# Supplementary material for: Diffusion MRI-based analysis of functional alterations of the glymphatic system in children with non-lesional epilepsy
Source: Neuroimage Rep. 2025 Jan 30;5(1):100237. doi: 10.1016/j.ynirp.2025.100237 (PMC12172819; doi:10.1016/j.ynirp.2025.100237)
Supplement: Multimedia component 1 [file mmc1.docx]

**Table S1 Information on brain regions with increased FW-WM in the NLE group**

| Cluster | Cluster  Voxels | Regions | Peak MNI | | | *P* value | Voxels |
| --- | --- | --- | --- | --- | --- | --- | --- |
|  |  |  | X | Y | Z |  |  |
| 1 | 22387 | Inferior Longitudinal Fasciculus R | 50 | -26 | -22 | 0.0020 | 1428 |
|  |  | Inferior Fronto-Occipital Fasciculus R |  |  |  |  | 1695 |
|  |  | Superior Longitudinal Fasciculus 3 R |  |  |  |  | 1454 |
|  |  | Superior Thalamic Radiation R |  |  |  |  | 1405 |
|  |  | Middle Longitudinal Fasciculus R |  |  |  |  | 1335 |
|  |  | Superior Longitudinal Fasciculus 2 R |  |  |  |  | 1104 |
|  |  | Corticospinal Tract R |  |  |  |  | 987 |
|  |  | Arcuate Fasciculus R |  |  |  |  | 981 |
|  |  | Anterior Thalamic Radiation R |  |  |  |  | 915 |
|  |  | Optic Radiation R |  |  |  |  | 864 |
|  |  | Frontal Aslant Tract R |  |  |  |  | 700 |
|  |  | Uncinate Fasciculus R |  |  |  |  | 664 |
|  |  | Vertical Occipital Fasciculus R |  |  |  |  | 594 |
|  |  | Forceps Major |  |  |  |  | 511 |
|  |  | Acoustic Radiation R |  |  |  |  | 366 |
|  |  | Superior Longitudinal Fasciculus 1 R |  |  |  |  | 301 |
|  |  | Anterior Commissure |  |  |  |  | 299 |
|  |  | Cingulum subsection: Temporal R |  |  |  |  | 180 |
|  |  | Forceps Minor |  |  |  |  | 157 |
|  |  | Fornix R |  |  |  |  | 115 |
|  |  | Cingulum subsection: Dorsal R |  |  |  |  | 41 |
|  |  | Cingulum subsection: Peri-genual R |  |  |  |  | 6 |
|  |  | - |  |  |  |  | 6285 |
| 2 | 17654 | Superior Longitudinal Fasciculus 3 L | -43 | 1 | 22 | 0.0112 | 1050 |
|  |  | Anterior Thalamic Radiation L |  |  |  |  | 1276 |
|  |  | Superior Longitudinal Fasciculus 2 L |  |  |  |  | 1119 |
|  |  | Superior Thalamic Radiation L |  |  |  |  | 1047 |
|  |  | Middle Longitudinal Fasciculus L |  |  |  |  | 1020 |
|  |  | Corticospinal Tract L |  |  |  |  | 933 |
|  |  | Inferior Fronto-Occipital Fasciculus L |  |  |  |  | 925 |
|  |  | Arcuate Fasciculus L |  |  |  |  | 917 |
|  |  | Optic Radiation L |  |  |  |  | 866 |
|  |  | Frontal Aslant Tract L |  |  |  |  | 781 |
|  |  | Uncinate Fasciculus L |  |  |  |  | 561 |
|  |  | Cingulum subsection: Dorsal L |  |  |  |  | 536 |
|  |  | Vertical Occipital Fasciculus L |  |  |  |  | 475 |
|  |  | Inferior Longitudinal Fasciculus L |  |  |  |  | 473 |
|  |  | Superior Longitudinal Fasciculus 1 L |  |  |  |  | 416 |
|  |  | Acoustic Radiation L |  |  |  |  | 333 |
|  |  | Forceps Major |  |  |  |  | 230 |
|  |  | Fornix L |  |  |  |  | 186 |
|  |  | Anterior Commissure |  |  |  |  | 170 |
|  |  | Forceps Minor |  |  |  |  | 170 |
|  |  | Cingulum subsection: Temporal L |  |  |  |  | 46 |
|  |  | Cingulum subsection: Peri-genual L |  |  |  |  | 19 |
|  |  | Corticospinal Tract R |  |  |  |  | 1 |
|  |  | - |  |  |  |  | 4104 |
| 3 | 1424 | - | -25 | -64 | 28 | 0.0392 | 743 |
|  |  | Superior Longitudinal Fasciculus 1 L |  |  |  |  | 304 |
|  |  | Superior Longitudinal Fasciculus 2 L |  |  |  |  | 169 |
|  |  | Forceps Major |  |  |  |  | 150 |
|  |  | Middle Longitudinal Fasciculus L |  |  |  |  | 50 |
|  |  | Cingulum subsection: Dorsal L |  |  |  |  | 7 |
|  |  | Inferior Fronto-Occipital Fasciculus L |  |  |  |  | 1 |
| 4 | 431 | Corticospinal Tract R | 20 | -34 | 52 | 0.0468 | 246 |
|  |  | Superior Longitudinal Fasciculus 1 R |  |  |  |  | 167 |
|  |  | - |  |  |  |  | 18 |
| 5 | 289 | Forceps Minor | -13 | 37 | -15 | 0.0448 | 352 |
|  |  | Uncinate Fasciculus L |  |  |  |  | 25 |
|  |  | Inferior Fronto-Occipital Fasciculus L |  |  |  |  | 3 |
|  |  | Cingulum subsection: Dorsal L |  |  |  |  | 1 |
|  |  | - |  |  |  |  | 8 |
| 6 | 180 | Anterior Thalamic Radiation L | -21 | 21 | 3 | 0.0476 | 159 |
|  |  | Inferior Fronto-Occipital Fasciculus L |  |  |  |  | 2 |
|  |  | - |  |  |  |  | 19 |
| 7 | 109 | Inferior Longitudinal Fasciculus L | -52 | -47 | -13 | 0.0314 | 108 |
|  |  | - |  |  |  |  | 1 |
| 8 | 107 | Corticospinal Tract | 29 | -19 | 55 | 0.0432 | 87 |
|  |  | - |  |  |  |  | 20 |
| 9 | 89 | Inferior Longitudinal Fasciculus R | 44 | 1 | -32 | 0.0214 | 88 |
|  |  | - |  |  |  |  | 1 |
| 10 | 82 | - | -10 | -68 | 39 | 0.0478 | 63 |
|  |  | Superior Longitudinal Fasciculus 1 L |  |  |  |  | 19 |
| 11 | 44 | Forceps Major | 21 | -48 | 9 | 0.0214 | 44 |
| 12 | 28 | Superior Thalamic Radiation L | -28 | -3 | 19 | 0.0128 | 17 |
|  |  | Corticospinal Tract L |  |  |  |  | 4 |
|  |  | - |  |  |  |  | 7 |
| 13 | 28 | Corticospinal Tract R | 12 | -26 | 62 | 0.0480 | 28 |
| 14 | 26 | - | -7 | -19 | 15 | 0.0482 | 2 |
|  |  | Superior Thalamic Radiation L |  |  |  |  | 24 |
| 15 | 21 | Inferior Fronto-Occipital Fasciculus L | -26 | 17 | 11 | 0.0480 | 11 |
|  |  | Frontal Aslant Tract L |  |  |  |  | 1 |
|  |  | - |  |  |  |  | 9 |

*Notes:* “-” indicates that the location of the corresponding regions is outside the XTRACT atlas.
